# Exploring High-Spin Color Centers in Wide Band Gap Semiconductors SiC: A Comprehensive Magnetic Resonance Investigation (EPR and ENDOR Analysis)

**DOI:** 10.3390/molecules29133033

**Published:** 2024-06-26

**Authors:** Larisa Latypova, Fadis Murzakhanov, George Mamin, Margarita Sadovnikova, Hans Jurgen von Bardeleben, Julietta V. Rau, Marat Gafurov

**Affiliations:** 1 School of Chemistry and Chemical Engineering, Harbin Institute of Technology, 92 West Da-Zhi Street, Harbin 150001, China; larisa.latypova@hit.edu.cn; 2 Zhengzhou Research Institute, Harbin Institute of Technology, 26 Intersection of Longyuan East 7th Street and Longhu Central North Road, Zhengdong New District, Zhengzhou 450046, China; 3 Institute of Physics, Kazan Federal University, Kremlevskaya 18, 420008 Kazan, Russia; georgemamin@gmail.com (G.M.); margaritaasadov@gmail.com (M.S.);; 4 Institut des Nanosciences de Paris, Campus Pierre et Marie Curie, Sorbonne Université, 4, Place Jussieu, 75005 Paris, France; vonbarde@insp.jussieu.fr; 5 Istituto di Struttura della Materia, Consiglio Nazionale delle Ricerche, ISM-CNR, Via del Fosso del Cavaliere 100, 00133 Rome, Italy; 6 Department of Analytical, Physical and Colloid Chemistry, Institute of Pharmacy, I.M. Sechenov First Moscow State Medical University, Trubetskaya 8, Build. 2, 119048 Moscow, Russia

**Keywords:** color centers, semiconductors, electron paramagnetic resonance, nitrogen-vacancy center, silicon carbide, electron–nuclear double resonance

## Abstract

High-spin defects (color centers) in wide-gap semiconductors are considered as a basis for the implementation of quantum technologies due to the unique combination of their spin, optical, charge, and coherent properties. A silicon carbide (SiC) crystal can act as a matrix for a wide variety of optically active vacancy-type defects, which manifest themselves as single-photon sources or spin qubits. Among the defects, the nitrogen-vacancy centers (*NV*) are of particular importance. This paper is devoted to the application of the photoinduced electron paramagnetic resonance (EPR) and electron–nuclear double resonance (ENDOR) techniques at a high-frequency range (94 GHz) to obtain unique information about the nature and properties of NV defects in SiC crystal of the hexagonal 4H and 6H polytypes. Selective excitation by microwave and radio frequency pulses makes it possible to determine the microscopic structure of the color center, the zero-field splitting constant (*D* = 1.2–1.3 GHz), the phase coherence time (*T*_2_), and the values of hyperfine (≈1.1 MHz) and quadrupole (*C*_q_ ≈ 2.45 MHz) interactions and to define the isotropic (*a* = −1.2 MHz) and anisotropic (*b* = 10–20 kHz) contributions of the electron–nuclear interaction. The obtained data are essential for the implementation of the *NV* defects in SiC as quantum registers, enabling the optical initialization of the electron spin to establish spin–photon interfaces. Moreover, the combination of optical, microwave, and radio frequency resonant effects on spin centers within a SiC crystal shows the potential for employing pulse EPR and ENDOR sequences to implement protocols for quantum computing algorithms and gates.

## 1. Introduction

The advancement of modern information and computing technologies has significantly enriched our comprehension of experimental phenomena across scientific disciplines, such as condensed matter physics and materials science [1,2]. However, the computing capabilities of contemporary processors are approaching their physical limits due to constraints imposed by nanoscale transistors and the finite speed of light [3,4]. In response to these challenges, a novel approach emerged in the early 2000s, leveraging logical operations rooted in the principles of quantum mechanics [5]. Quantum bits (qubits), serving as fundamental units of information, can be realized through the spins of electron or nuclei, photon polarization, or energy states of superconducting Josephson junctions or ultracold Rydberg atoms [6]. These systems exhibit two key properties: superposition and quantum entanglement [7].

Despite the demonstrated computational superiority of quantum simulators over classical counterparts in solving identical model problems, quantum technologies are still in their nascent stages, requiring substantial advancements in both the material infrastructure and understanding of quantum information processing algorithms [8,9,10,11,12]. Spin defects within semiconductor materials are promising candidates for quantum technologies [13]. Notable examples include the nitrogen-vacancy (${NV}^{-}$) center in diamond [14], vacancy centers within the silicon carbide (SiC) family [15], and the boron vacancy in a two-dimensional hexagonal boron nitride (hBN) crystal [16]. These systems share common characteristics, such as the presence of an energy level within the semiconductor band gap, a high-spin state (electron spin *S* = 1) with zero-field splitting (ZFS) *D*, and the potential for spin initialization through optical, microwave (MW), or radio frequency (RF) manipulations [13,17,18]. Luminescence spectra in the visible and near-infrared (IR) regions allow for designating them as color centers.

**Figure 1:**
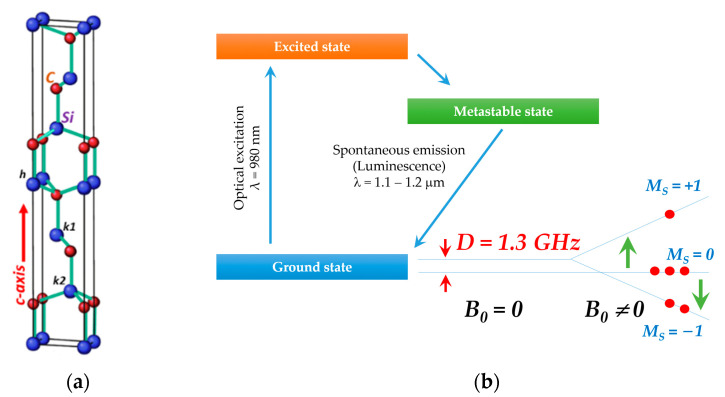
(**a**) The structure of a bulk crystal of silicon carbide of the hexagonal 6H polytype, comprising carbon atoms (red balls) and silicon (blue balls). Symbols h, k1, and k2 denote alternating structurally nonequivalent atomic positions. (**b**) The scheme illustrating the optical polarization of nitrogen-vacancy states by a laser source. Due to the mechanism of intersystem crossing during the transition of a defect from the excited orbital (^3^E) state to the ground (^3^A_2_) state, the spin sublevel is populated with *M*_S_ = 0 (*M*_S_ is the quantum number of the electron spin projection). The arrows indicate EPR transitions that generate the signal of absorption and emission (population inversion) of MW energy.

Silicon carbide stands out as a prominent semiconductor material in the realm of quantum technologies [19,20]. Despite the apparent simplicity of the chemical formula, the SiC crystals have many structural modifications (more than 250) [21]. Such a polytypism leads to variability in the position of atoms along the crystallographic axis *c* (2H, 3C, 4H, 6H, and 15R). Figure 1a shows the structure of the SiC of the hexagonal 6H polytype, which contains three structurally nonequivalent positions for each of the atoms, highlighted as k1, h, and k2. These positions differ in local interatomic distance and symmetry in the second coordination sphere, where h is hexagonal and k is quasi-cubic symmetry. SiC materials harbor a diverse range of electronic defects characterized by triplet state (electronic spin *S* = 1) and extended phase coherence times (determined by spin–lattice and spin–spin interactions), amenable to optical manipulation [22,23]. The luminescence of optically polarizable centers in SiC predominantly appears in the near-IR region, with zero-phonon luminescence lines aligning with the transparency windows of optical fibers (S- and O-bands for telecommunications) and biological entities (for biosensing) [20].

Electron paramagnetic resonance (EPR) techniques, including electron–nuclear double resonance (ENDOR), are essential tools for investigating the electronic and coherent properties of spin defects [24]. ENDOR provides deep insights into hyperfine and quadrupole spin–nuclei interactions, which are crucial for implementing multiqubit quantum registers, such as quantum memory [24,25]. The optical initialization of spin defects in SiC presents a unique opportunity for developing spin–photon interfaces with integrated electron and photonic qubits [26]. The optical polarization of high-spin defects with ZFS enables the detection of individual centers at room temperature, paving the way for highly sensitive temperature and magnetic field sensors with a submicron resolution [27,28,29].

The exploration and identification of electronic configurations with optically polarizable triplet states within the SiC crystal lattice represent an intriguing avenue for scientific research and a wide range of applications. In this work, the spin-optical, coherent, and electron–nuclear properties of *NV* centers in a SiC crystal of the hexagonal polytypes 4H and 6H were studied using the photoinduced EPR and ENDOR methods at the high-frequency MW range (W-band, 94 GHz).

## 2. Results and Discussion

### 2.1. Spin-Optical Properties

**Figure 2:**
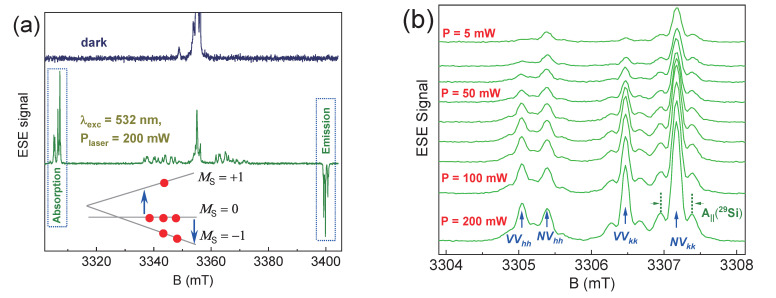
(**a**) The ESE EPR spectra at room temperature for the 4H-SiC sample, depending on the laser pumping and the triplet state scheme of the color center. Due to the mechanism of intersystem crossing during the transition of a defect from the excited (^3^E) to the ground (^3^A_2_) state, the spin sublevel with *M*_S_ = 0 becomes the most populated. The arrows in the low inset indicate EPR transitions that generate the signal of the absorption and emission (population inversion) of MW energy. (**b**) The detailed change in the low-field component of the EPR spectrum depending on the laser power. The shoulders arising for the NV centers and divacancies are associated with the presence of a hyperfine interaction with A_||_ = 4.5 MHz from the magnetic isotopes of ^29^Si.

The electron spin echo (ESE)-detected EPR spectrum (ESE EPR) for the 4H-SiC sample shown in Figure 2a contains a large number of signals that are sensitive to laser excitation. The specific charge state of defects in SiC (a variety of *VV* and *NV* centers) leads to the appearance of paramagnetic centers with *S* = 1. The dipole–dipole interaction between the paramagnetic centers and the gradient of the electric (crystal) field appear as a doublet in the EPR spectra. Laser pumping results in a predominantly spin population of *M*_S_ = 0, leading to a significant increase in the EPR signal and the observation of both absorption (low-field) and emission (high-field) resonance lines [30,31]. The mechanism responsible for optical polarization (photoactivation) is associated with intersystem crossing conversion during the optical transition from the ground ^3^A_2_ to the excited state ^3^E (Figure 1b). In this case, nonradiative recombination occurs through the intermediate metastable state ^1^A with a predominant population of the ground state with *M*_S_ = 0. To describe color centers with an electron spin *S* = 1, the following spin Hamiltonian is used:

(1) H=gμBB·S+DSz2−23+ESx2−Sy2+A||SzIz+A⊥SxIx+SyIy+PIz2−23

where *g* is the spectroscopic splitting factor, *μ*_B_ is the Bohr magneton, *D* and *E* are the ZFS values, and *S*_x,y,z_ and *I*_x,y,z_ are the projections of the electron and nuclear spin; *A* and *P* are the values of the hyperfine and quadrupole interactions.

Figure 2b displays the relationship between the intensity of low-field absorption lines and the power of the laser source. Notably, *NV* centers and divacancies *VV* exhibit distinct responses to optical pumping. Initially, *VV*_hh_ is barely discernible on the EPR spectrum, but it later becomes more pronounced than *NV*_hh_. The level of polarization of each defect center (*NV* or *VV*) is contingent on the activation energy and the probability coefficient (pre-exponential factor). As a result, the findings from the EPR spectra can offer insights into the energy depth of vacancy-type defects based on electronic band structure theory [32].

**Figure 3:**
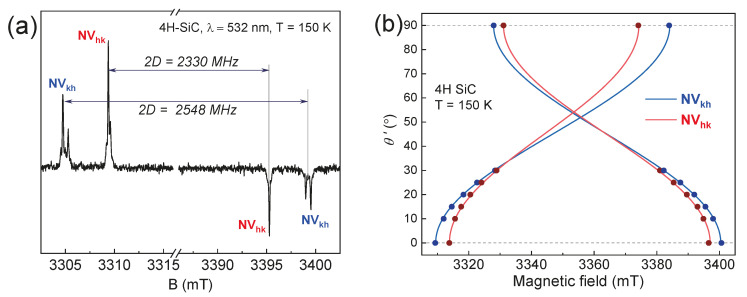
(**a**) The ESE EPR spectrum of the 4H–SiC for the basal *NV* centers (*θ* = 70° or *θ*′ *=* 0°) under laser excitation; (**b**) the angular variation *θ*′ *= θ* – 70° in the resonance lines of the basal NV centers for the rotation of the magnetic field in the (1120) plane.

Structurally, the basal center axes are rotated 70° degrees with respect to the axial defects (*NV*_hh_ and *NV*_kk_). Using a goniometer of the spectrometer, the SiC crystal was rotated 70 degrees from the parallel orientation, as shown in Figure 3a. This 70-degree orientation is regarded as canonical (*θ*′ *=* 0°) for basal centers and serves as the parallel direction for *NV*_kh_ and *NV*_hk_ centers [33]. The simulation of the experimental ZFS spectral lines (Figure 3b) using the spin Hamiltonian (1) parameters from Table 1 verifies that the observed centers are indeed basal centers.

**Figure 4:**
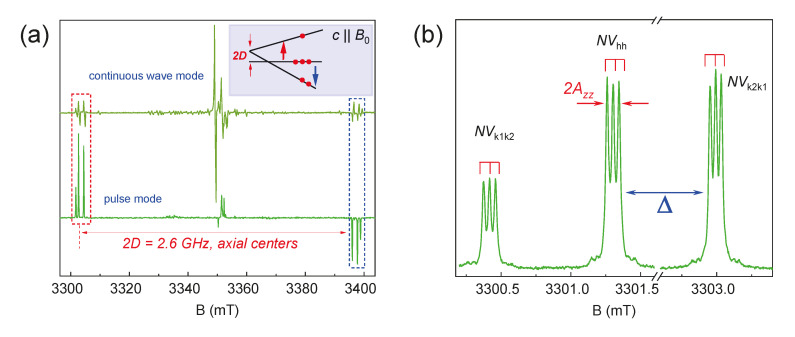
(**a**) The EPR spectra in the conventional continuous wave (top) and ESE (bottom) modes with the c-axis of the 6H-^28^SiC crystal oriented parallel to *B*_0_. The inset shows the spin levels of the defect with *S* = 1 under optical excitation at λ = 980 nm. (**b**) The detail of the low-field components highlighted by the red rectangle in (**a**). The experiments were carried out at *T* = 150 K.

In various SiC polytypes (3C, 4H, 6H, and 15R), two-point symmetry groups for color centers can be distinguished: basal centers with *C*_1h_ symmetry and axial centers with *C*_3v_ symmetry. In Figure 4a, the axial *NV* centers in 6H-SiC have a ZFS with *D* approximately equal to 1.3 GHz, and, accordingly, in the parallel orientation of the crystal relative to B_0,_ the splitting between lines is 2*D* = 93 mT (≈2600 MHz). Meanwhile, basal centers with lower symmetry have a split of the order of 45 mT (≈1260 MHz). The smaller value is because the main *z*-axis of the microscopic structure of the basal center is deviated by ≈70° relative to the crystallographic *c*-axis of the crystal. It was previously shown that basal centers, under optimal experimental conditions, have a *D* value of approximately the same order (1.25 GHz) as the axial ones [34]. Also, in the EPR spectra, one can notice that each ZFS component contains three lines, which are associated with three *NV* centers in different positions of the crystal (k1k2, hh, and k2k1) but with the same point symmetry group. Each of these three structurally nonequivalent positions of the *NV* centers differ slightly in the local environment of the second coordination spheres and the distance between the silicon vacancy and the nitrogen atom, which affects the value of *D* in the EPR spectra [35].

The EPR spectra of the color centers in the 6H-^28^SiC crystal under laser excitation contain signals from the defects of various types. The components of interest are highlighted by rectangles (Figure 4a), which are associated with negatively charged *NV* centers (*S* = 1 and *D* ≈ 1.3 GHz) [36]. Figure 4b shows the details of the low-field ZFS component. It consists of three groups of lines due to the presence of structurally nonequivalent *NV* center positions (k1k2, hh, and k2k1) in 6H-^28^SiC (see Figure 1). The spectral position difference between the centers *NV*_k1k2_ and *NV*_k2k1_ (denoted as Δ in Figure 4b) is 70 MHz (2.5 mT), which allows for the use of standard MW generators for quantum manipulation. Thus, in 6H-^28^SiC, one can potentially create three independent qubits. 

The presence of ^14^N magnetic nuclei with *I* = 1 near a silicon vacancy leads to the additional hyperfine splitting of EPR lines into three (2*I* + 1 = 3) components with *A*_zz_ = 1.1 MHz (Figure 4b). The obtained electron–nuclear coupling provides an opportunity to initiate nuclear magnetic resonance (NMR) transitions by applying RF irradiation to realize “CNOT”-type operations [37].

**Figure 5:**
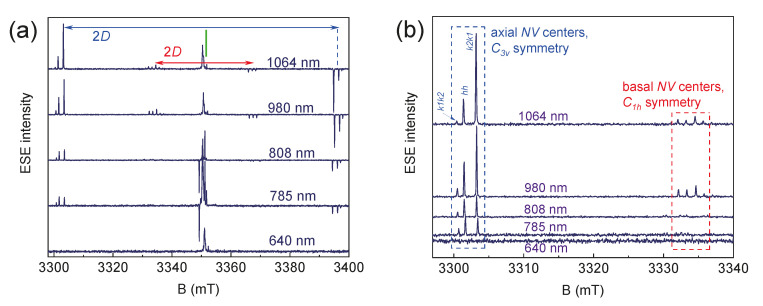
(**a**) ESE EPR spectra of color centers in 6H-SiC with full magnetic field sweep; (**b**) detailed view of low-field ZFS components for axial (blue rectangle) and basal (red rectangle) symmetry centers depending on the optical excitation wavelength. Experiments were carried out at *T* = 297 K.

No EPR signal was observed without optical irradiation. Figure 5 illustrates the dependence of the EPR spectra in the 6H-^28^SiC crystal on the wavelength. Effective spin polarization (inversion of the high-field component) is detected for the optical excitations with the wavelengths in the range from 785 nm to 1064 nm. Figure 5b demonstrates that optical excitation at λ = 980 nm leads to the spin polarization of *NV* centers of all types of symmetry and positions, while excitation at λ = 1064 nm results in a significant redistribution of intensities between the *NV*_k1k2_ and *NV*_k2k1_. The photoinduced EPR spectra at λ = 808 nm and λ = 785 nm contain weak signals only from the axial centers. This is likely due to the much lower probability of transitions between the orbital (optical) levels for basal centers [38]. 

The values of the optical excitations in the IR range with λ = 980 nm and λ = 1064 nm are close to the luminescence of the color centers of 1.1–1.2 μm [35]. The highest optical polarization was established for λ = 980 nm. The use of other wavelengths results only in a slight change in the EPR signal magnitudes without phase inversion. Ultraviolet radiation (λ = 260 nm) can lead to a change in the charge state (−1/0) of the color centers with *S* = 0, which are EPR-silent. The use of λ = 260 nm and 640 nm due to a larger energy quantum (4.77 eV and 1.94 eV, respectively) leads to a “transfer” of the center to higher excited orbital levels close to the conduction band, which are outside the optimal optical absorption region of the material. 

This result opens up new possibilities and prospects for integrating qubit structures for quantum technologies based on *NV* centers in 6H-SiC with existing fiber-optic electronics for the ultimate combination of quantum, electrical, and optical methods for high-speed information processing, transmission, and storage. In addition, it shows the suitability of the application of *NV* centers in 6H-SiC as sensitive biosensors for studying biological objects (with the bandwidth also in the near-IR region [20]).

**Figure 6:**
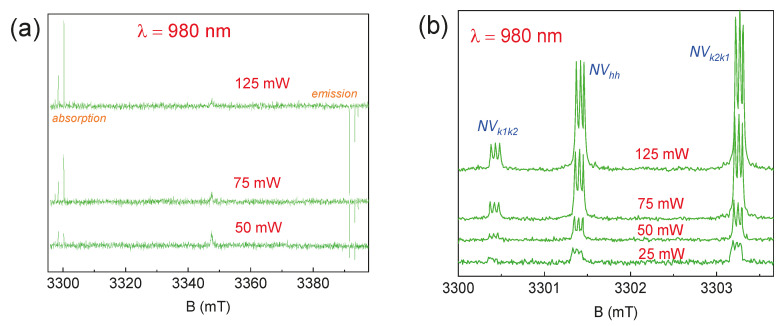
The ESE EPR spectra for 6H-SiC depending on the power of the laser source: (**a**) the full magnetic field sweep; (**b**) the detail of the low-field components. The experiments were carried out at *T* = 297 K.

Figure 6 shows the relationship between the output power of the laser source and the EPR signal. It was revealed that, under the specified experimental parameters, the values *P* = 25 and 125 mW represent the threshold for observing photoinduced EPR spectra. At *P* = 25 mW, the signal from *NV* centers is almost comparable to the noise level, and only a significantly larger number of accumulations makes it possible to detect defects. A further decrease in power leads to a complete loss of signal from the centers under study. With an increase in laser source power, a proportional increase in the EPR signal occurs, as was previously shown for boron vacancies in hBN [39]. With a further increase in *P* (higher than 125 mW), the spin system becomes saturated, leading to a sharp drop in the intensity of the EPR signal. A negative consequence of using high optical power is the local heating of the crystal, which leads to a decrease in electronic relaxation times. 

**Figure 7:**
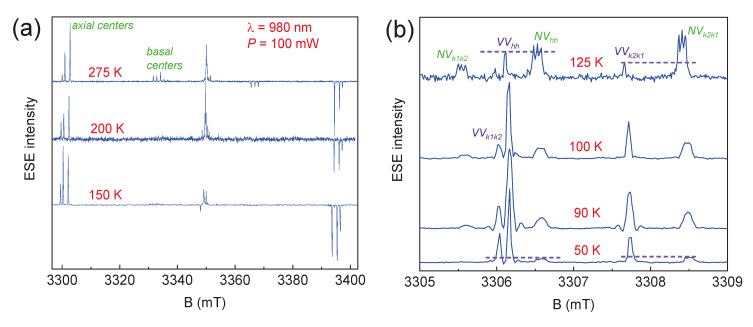
The ESE EPR spectra depending on the temperature of the 6H-SiC crystal under continuous excitation by a laser source with λ = 980 nm and *P* = 100 mW: (**a**) the full magnetic field sweep; (**b**) detail of low-field components.

Figure 7 shows the temperature dependences of the photoinduced EPR spectra. The EPR signals from the described above *NV* centers were observed up to *T* = 297 K, which is an important part of future studies of spin qubits for quantum technologies at room temperature [40,41,42,43]. Additional signals were detected at *T* ≤ 125 K, which were attributed to centers consisting of a silicon vacancy and a carbon vacancy, called divacancies (*VV*, see Figure 7b) [41]. Divacancy appearance leads to the significant intensity decreasing in EPR signals from the *NV* centers. This effect may be associated with the charge transfer between closely localized *VV* and *NV* color centers, leading to the change in the spin state of the *NV* center to the non-magnetic singlet state (*S* = 0).

### 2.2. Relaxation Characteristics

The spin relaxation and decoherence processes are frequently the main limiting factors in quantum technology applications. Pulse EPR techniques allow for measuring the various so-called dynamical parameters of the spin systems, which describe these processes. In this subsection, the results of the determination of the spin–lattice (longitudinal) relaxation time *T*_1_ extracted from the inversion-recovery pulse sequence and the phase coherence time *T*_2_ from the Hahn sequence as well as other time parameters are presented. The details of their measurements are given in Section 3.

**Figure 8:**
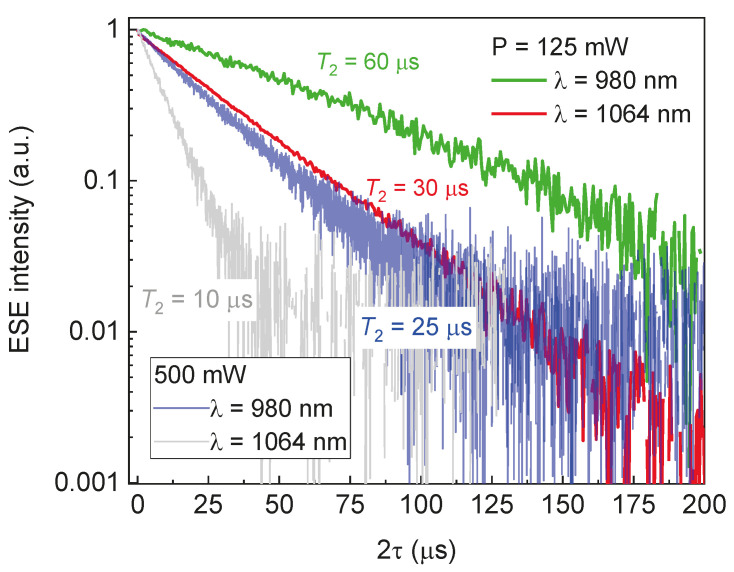
The decay curves of the transverse magnetization (*T*_2_) of the *NV* center in 6H-SiC on a semi-logarithmic scale at *T* = 150 K under the influence of optical excitation with *P* = 500 mW (blue and gray lines) and 125 mW (red and green lines) at λ = 980 nm (blue and green lines) and 1064 nm (red and gray lines).

Figure 8 shows the *T*_2_ relaxation curves for the *NV*-spin defects in 6H-SiC under the influence of continuous light radiation at λ = 980 nm and 1064 nm with *P* = 500 mW and *P* = 125 mW. The phase coherence decay is caused by spin–spin interactions between *NV* centers and spin diffusion, which manifests itself in the deviation from the straight line [44]. Reducing the average laser power by a factor of 4 led to an increase in the phase coherence time by two times. For *T* = 150 K, λ = 980 nm, and *P* = 125 mW, one extracts *T*_2_ = 60 µs, which is longer than reported previously for the vacancy defects even at a lower temperature (for the boron vacancy in hBN: *T*_2_ =15 µs [16], and for the divacancies in 4H-SiC: *T*_2_ = 40 µs at *T* = 7 K [45]). Thus, due to their excellent coherent properties, the *NV* center in 6H-^28^SiC can be regarded as a suitable platform for the implementation of quantum information technologies based on spin–photon interactions.

**Figure 9:**
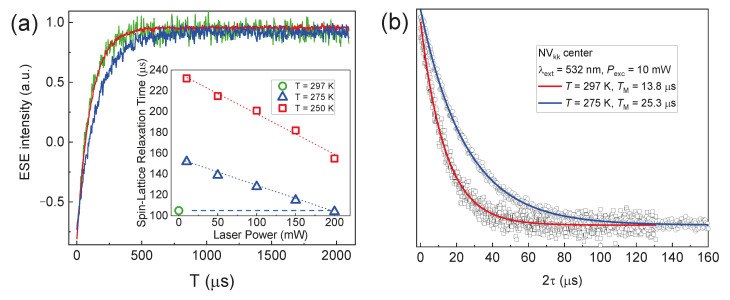
(**a**) The recovery magnetization curves (*T*_1_) for NV centers in 4H-SiC depending on the SiC temperature of 297 (green curve, red line) and 275 K (blue curve) at *P* = 10 mW. The insert shows the *T*_1_ times for various laser powers with λ = 980 nm; (**b**) phase coherence decay (*T*_2_) curves depending on the crystal temperature. The experimental curves are fitted by single exponential functions with the listed parameters of *T*_1_ and *T*_2_.

The results for 4H-SiC are as follows. Initially, at room temperature (297 K), the laser radiation power with λ = 532 nm was set at its minimum level (*P* = 10 mW) for a duration of 10 s, following which it was turned off to record the “dark” signals. Under these experimental conditions, *T*_1_ = 106(3) µs and *T*_2_ = 13.8 µs for the *NV*_kk_ centers were extracted (Figure 9a, green curve; Figure 9b, red line, respectively). Notably, at room temperature, *T*_2_ is twice as long as that measured in van der Waals hBN crystals at *T* = 50 K (*T*_2_ = 15 µs) [16], highlighting the potentials for using color centers in 4H-SiC as the base for the room temperature quantum technologies. At 275 K, the longer values *T*_1_ = 152(2) µs and *T*_2_ = 25.3 µs for the *NV*_kk_ centers were estimated (blue curves in Figure 9a and Figure 9b, respectively). Figure 9a (inset) also illustrates a linear correlation between the *T*_1_ and laser source power, indicating the prevalence of the “direct relaxation process” for *NV* centers [46]. 

It should be noted that the phase coherence decay curves in the investigated species follow a single exponential pattern, which is a sign of the dominant spin–spin relaxation mechanism without the spin (spectral) diffusion contribution [45]. The temperature measurements indicate an impact of the spin–lattice relaxation on the phase coherence time according to the formula 1/*T*′_2_ = 1/*T*_2_ + 1/*T*_1_.

A detailed study of the dynamic properties of the basal centers was conducted for the parallel orientation of the 4H-SiC crystal (*c* || B) to minimize the effects of cross-relaxation or nuclear diffusion, which arise due to the mixing of the wave functions in intermediate orientations [47]. The temperature *T* = 150 K was set as optimal: at this temperature, the measured *T*_1_ are long enough (*NV*_kh_ *T*_1_ = 1.43 ms and *NV*_hk_ *T*_1_ = 1.42 ms) and do not have a strong accelerating effect on *T*_2_. Thus, we can measure the *T*_2_ time that is largely caused by the spin–spin interaction between the defects. 

**Figure 10:**
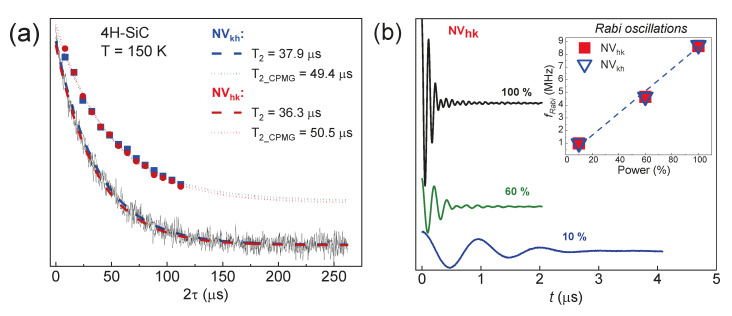
The dynamic characteristics of the basal centers for the parallel orientation of the 4H-SiC crystal: (**a**) a comparison of the transverse magnetization decay curves obtained by using the Hahn pulse sequence and the CPMG approach for each basal defect; (**b**) the Rabi oscillation curves of the center, depending on the supplied MW power with a frequency of 94 GHz. The insert shows the dependence of the Rabi oscillation frequency on the MW source power.

Initially, the *T*_2_ relaxation curve (λ = 532 nm and *P* = 10 mW) was measured by the usual Hahn pulse sequence. Then, an experiment was carried out by the Carr–Purcell–Meiboom–Gill (CPMG) sequence (see Section 3), which allows for eliminating the influence of the spin diffusion of various types on the coherence time [48]. A comparative analysis with the corresponding times for each type of basal center is shown in Figure 10a. The *T*_2_ values from the CPMG experiments are longer than those from the Hahn echo ones, demonstrating among other things that playing with the pulse sequences allows for manipulating spin systems with varying degrees of impact. 

It is worth noting that the detection of an ESE signal and long relaxation times do not necessarily enable the performance of multipulse experiments for manipulating the spin magnetic moment of the color center. The demonstration of the Rabi oscillations (see Section 3) is a crucial factor in enabling the utilization of promising centers for quantum technologies [49]. Figure 10b illustrates the Rabi oscillations for the basal NV centers. The oscillation frequency is determined by the gyromagnetic ratio constant value and the magnitude of the MW magnetic field component B1 in the EPR cavity. The inset of Figure 10b shows that the value of the Rabi oscillation frequency linearly depends on the MW power.

The linear frequency response of the electron magnetization suggests that Rabi oscillations arise solely from the influence of the MW pulse. The main mechanisms of dephasing, in addition to spin–spin relaxation, are the spread of local magnetic fields due to a dipole–dipole interaction and the inhomogeneous magnetic field B_1_ in the spectrometer cavity. The presence of these factors leads to the distribution of Rabi oscillation frequencies of the *NV* center within the sample. The linear dependence indicates the absence of two-quantum transitions (forbidden by the selection rule) between spin levels. Thus, the detection of long-term Rabi oscillations indicates the potential possibility of using the color centers as a qubit.

The experimental results described in this section demonstrate that the ensemble of axial *NV* centers in SiC possess sufficiently long relaxation times. This enables the detection of resonance signals in pulsed mode both under traditional low-temperature and ambient room conditions. The observed Rabi oscillations confirm that these color centers meet the criteria for spin manipulation, qualifying them as robust electron qubits.

### 2.3. Electron–Nuclear Interactions (Axial and Basal Centers)

To determine the terms of the spin Hamiltonian (1) and confirm the microscopic model for the *NV* center in 4H-SiC, ENDOR experiments were conducted. By applying MW and RF irradiations, it is possible to register NMR transitions indirectly through the transitions between the electronic sublevels of the electron–nuclear system. NMR transitions lead to a redistribution of the electron population and to a change in the integral intensity of EPR absorption. As a result, the components of ENDOR spectra are observed in the vicinity of the Larmor frequency of the magnetic nuclei [47]. 

**Figure 11:**
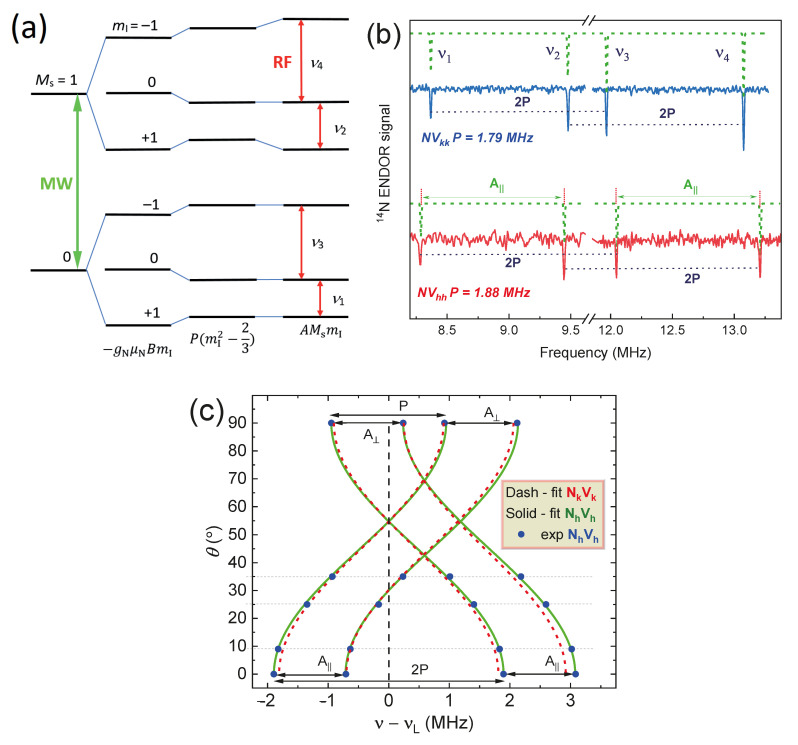
(**a**) The energy levels of the nitrogen-vacancy centers in accordance with the spin Hamiltonian (1) terms; (**b**) the ENDOR spectra at the parallel orientation of 4H-SiC crystal (*c* || B) for two different types of paramagnetic defects (*NV*_kk_ and *NV*_hh_) in a low-field ZFS component (*M*_S_: 0 → +1), which shows a visible difference in the parameters of the hyperfine *A* and quadrupole *P* interactions. The experimental data are supported by a theoretical simulation (dash lines); (**c**) the angular dependence of the ENDOR line positions for the *NV*_kk_ (solid curves) and *NV*_hh_ (dashed curves) due to the presence of a quadrupole interaction, as well as a weak anisotropic dipole–dipole hyperfine interaction.

The corresponding quadrupole and hyperfine interactions of the spin defects in 4H-SiC and the nuclear moments of ^14^N (*I* = 1) lead to energy splitting (Figure 11a) and the formation of four narrow lines in the region of the ^14^N Larmor frequency ν ≈ 10.2 MHz (*B*_0_ = 3.4 T). Figure 11b displays the ENDOR lines alongside a comparative analysis of the results for a similar axial type of *NV*_kk_ center in 4H-SiC crystal. The observed differences in the quadrupole splitting values (*P*) indicate variations in the nature (spin density distribution) and local environment of *NV* defects [45]. In order to determine the values of electron–nuclear interactions precisely, ENDOR spectra were acquired depending on the orientation of the SiC crystal relative to the magnetic field. A comparative analysis with the previous results for the *NV*_kk_ center and the simulation of experimental data for *NV*_hh_ allows for extracting the values of the isotropic and anisotropic contributions to the hyperfine interaction, as well as the value of the quadrupole splitting constant [47]. It should be noted that $A_{\left|\right|}=a+2b$ (parallel) and $A_{⊥}=a-b$ (perpendicular orientation), where *a* and *b* represent the isotropic and anisotropic values. Based on the ENDOR results obtained, the macroscopic model of the center under study was unambiguously established, where (i) *NV*_kk_ *A*_||_ = 1.11 MHz and *P* = 1.79 MHz (*C*_q_ = 2.39 MHz—quadrupole coupling constant) and (ii) *NV*_hh_ *A*_||_ = 1.16 MHz and *P* = 1.88 MHz (*C*_q_ = 2.51 MHz).

**Figure 12:**
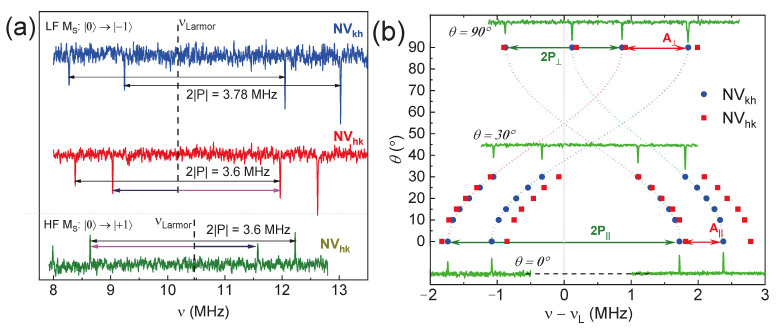
(**a**) The ENDOR spectra of the 4H-SiC for the basal *NV* centers (*θ* = 70° from c-axis) under laser excitation of 532 nm depend on the spin transition ZFS component. LF—low-field (*M*_S_: 0 → +1) and HF—high-field (*M*_S_: 0 → −1) transitions; (**b**) the angular dependence of the ENDOR line positions for the *NV*_kh_ (blue circle) and *NV*_hk_ (red square) due to the presence of a quadrupole interaction, as well as a weak anisotropic dipole–dipole hyperfine interaction.

The methodology for analyzing electron–nuclear coupling focuses on investigating centers with axial symmetry. ENDOR experiments were also conducted to probe the energy interactions of individual basal centers and to quantify the corresponding hyperfine and quadrupole splitting constants. Due to the presence of nitrogen-centered defects, it is anticipated that four splitting lines will be observed (Figure 12a). A comparative evaluation is presented for basal defects in their standard parallel alignment, encompassing both the low-field EPR line and the high-field components of the ZFS, with a demonstration of the signal symmetry relative to the ^14^N Larmor frequency. The angular dependence measurements conducted selectively (Figure 12a) serve two primary purposes: firstly, to improve the accuracy in determining the quadrupole and hyperfine constants (including the isotropic and anisotropic contributions) (see Table 2) and, secondly, to clearly illustrate the differences between the two defects.

The diverse array of structurally distinct *NV* centers in a 4H-SiC crystal provides a significant advantage for constructing multiqubit quantum registers. This multiplicity enables the selective excitation and readout of the spin system state, facilitating the development of advanced quantum computing architectures [50]. The magnitudes of hyperfine (isotropic Fermi interaction and dipole–dipole contributions) and quadrupole interactions of all four structurally nonequivalent *NV* centers in the 4H-SiC crystal are listed in Table 2. The narrow (5–6 kHz) and well-resolved ENDOR resonance lines of the *NV* centers enable the precise and selective excitations necessary for the individual readout of spin states (qubits) in quantum technologies [51].

The interaction between electrons and nuclei is crucial in utilizing color centers as quantum registers, allowing for the creation of multi-level spin systems and the implementation of sophisticated quantum registers. Optically initialized spin defects with bound magnetic nuclei serve as a promising foundation for quantum technologies. Spin polarization resulting from optical excitation facilitates the establishment of “spin–photon” interfaces, while the transfer of magnetization from the electronic to the nuclear subsystem addresses the challenge of maintaining long-lived quantum memory [17,52]. Therefore, the detailed examination of hyperfine and quadrupole parameters (Table 2) is pertinent to quantum information technologies leveraging color centers surrounded by nuclear spins.

## 3. Materials and Methods

The 4H-SiC sample studied in this work was a commercial N-doped (2 × 10^17^ cm^−3^) 4H-SiC single crystal (n-type semiconductor). It was irradiated at *T* = 295 K with 12 MeV protons to a total fluence of 1 × 10^16^ cm^−2^ in order to create Si-vacancy centers. The sample was then annealed at *T* = 900 °C to allow for the formation of *V*_Si_*N*_C_ complexes by Si-vacancy diffusion. The sample size was 0.8 mm × 0.4 mm × 0.2 mm.

The semiconductor crystal 6H-SiC, with isotopic enrichment in ^28^Si nuclei (*I* = 0) to reduce the influence of the nuclear subsystem on the processes of phase coherence of the ensemble of electron spins, was grown by high-temperature sublimation from the gas phase (physical vapor transport, PVT) with the preliminary use of a precursor enriched with the ^28^Si isotope (up to ≈99%). The content of the ^29^Si isotope (*I* = 1/2) in the grown samples was determined by the EPR method and was about 1%, which is more than four times less than its natural abundance (4.67%) [29]. The concentration of the impurity nitrogen in the crystal was equal to *C* ≈ 10^17^ cm^−3^. In order to create the vacancy centers, the 6H-^28^SiC samples were irradiated with electrons with an energy of 2 MeV and a nominal dose of 4 × 10^18^ cm^−2^. To form stable negatively charged nitrogen–vacancy complexes, the crystal after irradiation was annealed at a temperature of *T* = 900 °C in an argon atmosphere for 2 h. The sample size was 0.45 mm × 0.45 mm × 0.67 mm. 

The magnetic resonance experiments were carried out by means of a multifunctional Elexsys E680 commercial spectrometer (Bruker, Karlsruhe, Germany) operated at 94 GHz (W-band). The experiments were conducted in continuous wave mode with MW irradiation of the sample using a microwave power of *P_MW_* = 2 mW. This power level was determined to be optimal for avoiding saturation of the EPR signal while maintaining an acceptable signal-to-noise ratio across the investigated temperature range. The amplitude and field-modulation frequency were set at 0.1 mT and 100 kHz, respectively. The exact operating frequency for microwave detection was approximately 93.986 GHz. To ensure accurate recording of the EPR spectra at this frequency, an automatic frequency control system was employed.

In the pulse mode, the EPR spectra were recorded by detecting the amplitude of the primary electron spin echo (ESE) as a function of the magnetic field sweep *B* using a pulse sequence π/2 − τ − π − τ − *ESE*, where durations of π/2 and π pulses were equal to 40 ns and 80 ns, accordingly. The interval between two MW pulses was τ = 240 ns. Short nanosecond-scale microwave pulses required a 1 kW amplifier to achieve a 90- or 180-degree spin magnetization turn in a rotational coordinate system. The pulse sequences were specified and configured using the EasyPanel 2.0 and Advanced modes embedded into the software of the spectrometer, allowing one to accurately optimize the pulse durations, intervals, and integration areas of the ESE with 4 ns steps. To achieve undistorted and saturated EPR signals at each temperature and a distinct color center, the short repetition time (SRT) was continuously adjusted, directly affecting the registration rate of one scan. The relaxation curves were measured with standard pulse sequences. The Hahn sequence (π/2 − τ + *dt* − π − τ *+ dt* − *ESE*) with an increasing interpulse gap τ′ = τ + *dt* in *dt* = 64 ns steps was applied to derive the phase coherence time *T*_2_. The inversion-recovery sequence (π − *T* + *dT* − π/2 − τ − π − τ − *ESE*, where *T* = 1.5 µs and *dT* = 1 µs) was used to estimate the spin–lattice (longitudinal) relaxation time *T*_1_. The registration of the transverse magnetization decay by the CPMG method was carried out through two detection channels (*X* and *Y*) using a pulse sequence: π*_X_*/2 − τ_1_ − (π*_Y_* − τ_2_ − π*_Y_*)_14_ − τ − *ESE*, where τ_1_ = 320 ns and τ_2_ = 4 μs. The Rabi oscillations were obtained by using a three-pulse sequence ϑ_Rabi_ − *T* − π/2 − τ − π − τ − *ESE*, where the first pulse, conventionally denoted by the symbol ϑ, varies from 4 ns to 7–10 µs with a step of 8 ns. The π/2 and π pulse durations were kept as 40 and 80 ns, correspondingly, during the study of the dynamic characteristics. The ENDOR spectra were obtained utilizing the Mims pulse sequence (π_MW_/2 − τ − π_MW_/2 − π_RF_ − π_MW_/2 − τ − *ESE*) with a 150 kW RF generator, where π_MW_ = 72 ns and π_RF_ = 18 µs. A satisfactory signal-to-noise ratio was ensured by the multi-scan recording (1024–4096 scans) of the ENDOR spectrum within a reasonable period (30 min–2 h). In all the experiments in the pulsed mode, the width of the rectangular pulse in the frequency range was 12.5 MHz (or 4.5 G), which was sufficient for selective resonant excitation. The low-temperature measurements were conducted by using a flow helium cryostat from Oxford Instruments. The samples were photoexcited by solid-state lasers (*λ* = 532, 640, 785, 808, 980 and 1064 nm) with an output power up to 500 mW. Photoexcitation of the color centers during the experiment was achieved using an optical fiber integrated into a leak-protected sample holder. This setup enabled the simultaneous exploitation of optical pumping with MW or RF sources without attenuation.

## 4. Conclusions

The spin-optical and coherence properties of negatively charged nitrogen-vacancy centers in silicon carbide crystals of 4H and 6H polytypes were studied using W-band photoinduced EPR/ENDOR spectroscopy. The spin polarization of vacancy defects was explored as a function of the optical excitation wavelength, output power, and crystal temperature. Thus, for *NV* centers in the 4H polytype, the optimal excitation wavelength is 532 nm (visible range, green color), whereas for 6H, λ = 980 nm (near-IR range). Pulsed EPR measurements for the obtained color centers can be recorded at room temperature with *T*_2_ = 25 μs, thereby opening new opportunities for quantum technologies in ambient conditions. Rabi oscillations of the electronic magnetization for *NV* centers were observed, confirming that the color centers in SiC meet the criteria for qubits in terms of spin manipulation. Studying the orientation dependence of the ENDOR lines enabled the accurate determination of the components of the isotropic contact Fermi and anisotropic dipole-dipole interactions. The full spin Hamiltonian of the axial and basal color centers was established, namely, the values of the *g*-factor, the zero-field splitting constant (*D* = 1.2–1.3 GHz), and the values of hyperfine (≈1.1 MHz) and quadrupole (*C*_q_ ≈ 2.45 MHz, axial symmetry) interactions, and the isotropic (*a* = −1.2 MHz) and anisotropic (*b* = 10–20 kHz) contributions of the electron–nuclear interaction.

The results obtained from the studies on electron–nuclear interactions are crucial for developing quantum registers, while optical initialization of the electron spin will enable the establishment of spin–photon interfaces. The combination of optical, microwave, and radio frequency resonant effects on spin centers in a SiC crystal demonstrates the potential to use multipulse sequences (MW and RF) to implement protocols for quantum computing algorithms and gates.

## Figures and Tables

**Table 1:** The spin Hamiltonian EPR parameters of the axial and basal centers in the SiC crystals.

	*g*_⊥_	*g*_||_	*D* (MHz)	*E* (MHz)
*NV*_kk_	2.001(1)	2.004(1)	1299(10)	0
*NV*_hh_	2.003(1)	2.004(1)	1349(10)	0
*NV*_kh_	2.001(1)	2.003(1)	1274(20)	13(2)
*NV*_hk_	2.003(1)	2.002(1)	1165(20)	110(5)

**Table 2:** Hyperfine (isotropic and dipole–dipole contribution) and quadrupole splitting values from ENDOR measurements of basal centers.

	*P* (MHz)	*C*_q_ (MHz)	*a* (Isotropic, MHz)	*b* (Anisotropic, kHz)
*NV*_kk_	1.81	2.413	−1.14	14
*NV*_hh_	1.895	2.53	−1.185	10
*NV*_kh_	1.82(2)	2.43(1)	−1.05	4
*NV*_hk_	1.73(2)	2.31(1)	−0.87	11

## Data Availability

The data can be made available upon a reasonable official request to the corresponding authors.
